# Low-Dimensional Palladium on Graphite-on-Paper Substrate for Hydrogen Sensing

**DOI:** 10.3390/s22103926

**Published:** 2022-05-22

**Authors:** Boyi Wang, Takeshi Hashishin, Dzung Viet Dao, Yong Zhu

**Affiliations:** 1 School of Mechanical and Electronic Engineering, Wuhan University of Technology, Wuhan 430070, China; boyi.wang@whut.edu.cn; 2 Faculty of Advanced Science & Technology, Kumamoto University, 2-39-1 Kurokami, Kumamoto 860-8555, Japan; hashishin@msre.kumamoto-u.ac.jp; 3 Queensland Micro- and Nanotechnology Centre, Griffith University, Nathan, QLD 4111, Australia; d.dao@griffith.edu.au

**Keywords:** palladium, graphite on paper, hydrogen sensor, low-dimensional, microfibers networks

## Abstract

To stabilize the detection signal of palladium-based hydrogen sensors on paper substrates, a graphite intermediate layer was painted on the surface of paper. The graphite-on-paper (GOP) substrate offers advantages such as good thermo-electrical conductivity, low cost, and uncomplicated preparation technology. Quasi-1-dimensional palladium (Pd) thin films with 8 nm and 60 nm thicknesses were deposited on the GOP substrates using the vacuum evaporation technique. Thanks to the unique properties of the GOP substrate, a continuous Pd microfiber network structure appeared after deposition of the ultra-thin Pd film. Additionally, the sensing performance of the palladium-based hydrogen sensor was not affected, whether using GOP or paper substrate at 25 °C. Surprisingly, heating-induced loss of sensitivity was restrained due to the increased electrical conductivity of the GOP substrate at 50 °C.

## 1. Introduction

Hydrogen (H_2_) gas has a wide range of applications, including in spaceships, commerce, propulsion systems [1], H_2_ fuel cells [2], and H_2_-fueled cars [3]. Furthermore, it is also widely used in scientific research and industrial manufacturing. As a clean, economical, and environmentally friendly energy carrier, H_2_ has the potential to replace fossil fuels [4]. Hence, the problems of air pollution and greenhouse gas emissions can be addressed. Although H_2_ is a clean form of energy in our daily life, it explodes when its concentration is over 4% in air. Due to the properties of H_2_, leaked H_2_ cannot be detected by human senses. To use H_2_ with safety, a rapid, economical, reliable, and feasible H_2_ detection and measurement device is generally required for all H_2_-based applications. Typically, the devices available on the market that have been employed in H_2_ detection and measurement are metal-oxide-based H_2_ sensors [5]. However, these H_2_ sensors do not present the same superior sensing performance as palladium (Pd)-based H_2_ sensors [6,7,8,9,10]. 

Pd and its alloys have been reported as being excellent sensing materials for H_2_ detection and measurement, presenting a higher sensitivity to and selectivity for H_2_ at room temperature. While Pd alloys show better properties with respect to H_2_ sensing and inhibition of α to β phase transition [7], the alloy deposition process is complicated and costly. To achieve higher H_2_ sensing performance, pure Pd H_2_ sensors with a low-dimensional structure have been investigated for H_2_ leakage detection and concentration measurement [4].

Low-dimensional pure Pd H_2_ sensors have been a significant subject for research and application in the last few decades. Because they can provide a larger surface area-to-volume ratio and shorter diffusion path, the response time, recovery time, and gas response of these H_2_ sensors can be improved significantly. Recently, various low-dimensional Pd H_2_ sensors have been reported by several researchers. Kim et al. [11] demonstrated that a nanoporous Pd film was formed using anodic aluminum oxides (AAOs) template with a microelectromechanical system (MEMS) fabrication technique. The unique template allowed the deposited Pd thin film to have a larger surface area-to-volume ratio than a flat Pd thin film, which led to improved sensing performance for the H_2_ sensor. Although it had the advantage of a large surface area-to-volume ratio, the response time was unsatisfactory, and the gas response was lower at increased detection temperatures. As a further development of low-dimensional Pd H_2_ sensors, a single Pd nanowire was reported by Offermans et al. [12]. This H_2_ sensor showed a faster response and higher gas response when exposed to H_2_. While the lower-dimensional Pd nanowire provided a larger surface area-to-volume ratio than the thin film, the fabrication was complex and uncontrollable. The research group of Zeng et al. [1,9] focused on investigating networks of ultrasmall Pd nanowires on filtration membranes. This particular Pd nanowire network structure was easily formed after deposition of Pd on the surface of the expensive filtration membranes. Because the Pd ultra-thin film was directly deposited on the rough surface, the broken Pd nanowires resulted in an unstable sensing performance when exposed to high H_2_ concentrations. Therefore, a chromium (Cr) layer was deposited in between the substrate and the Pd thin film to modify the Pd–substrate interaction, which in return reduced the critical thickness of the Pd layer required to form a continuous Pd nanowire network [1]. Although the response speed of the H_2_ sensor was improved by decreasing the thickness of the Pd film and increasing the temperature, heating-induced loss of sensitivity still existed, as reported by Li et al. [13]. The solubility of H in Pd was reduced at increased temperatures, resulting in sensitivity disparities between the sensor at room temperature and at high temperature.

In this paper, unstable sensing performance of the ultra-thin Pd film was observed when 8 nm Pd was coated directly onto the paper substrate. To address this issue, we proposed to introduce a graphite layer between the Pd thin film and the paper substrate. Based on the surface texture of the GOP substrate, ultra-thin (≤10 nm) and thin (≤100 nm) continuous Pd microfiber networks were formed by using a conventional vacuum evaporation technique. The surface morphology of the quasi-1-dimensional (1.5D) Pd sensing layer was clearly observed using a scanning electron microscope (SEM). The effect of the intermediate graphite layer on the H_2_ sensing performance of the sensor at different detection temperatures is evaluated and determined in this study. Additionally, heating-induced loss of sensitivity for 60 nm Pd film at 50 °C was restrained.

## 2. Materials and Methods

### 2.1. Sensor Fabrication

It is well known that paper (A4/80gsm, Paper Australia Pty Ltd, Victoria, Australia) possesses several advantages, including low cost, light weight, and ubiquity. Hence, as the substrates of the H_2_ sensors in this study, these advantages can contribute to the investigation and development of Pd-based H_2_ sensors. A thin graphite layer was painted on the surface of the paper to produce a GOP substrate. Subsequently, pure Pd layers with thicknesses of 8 nm and 60 nm were deposited on the surface of the GOP substrates. The physical properties of graphite allow it to be coated onto paper substrates by hand without damaging the surface texture. Therefore, the 1.5D structure of the Pd microfiber network can be formed on the substrate following the deposition process, which in return results in a larger surface area-to-volume ratio. In this context, the H_2_ sensor can be improved with respect to its sensing performance. 

**Figure 1:**
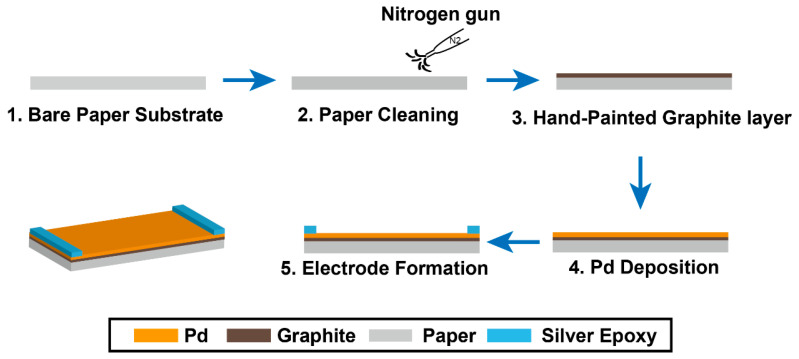
Schematic illustration of the fabrication steps of the palladium (Pd) microfiber network H_2_ sensor based on graphite-on-paper (GOP) substrate.

The simple step-by-step fabrication process of the H_2_ sensor is briefly depicted in Figure 1. The most crucial step before the sensor fabrication process is to clean and remove the surface dust on the paper using a high-pressure nitrogen gun (Figure 1 (2)). To acquire a homogeneous graphite layer on the surface of paper, it is necessary to paint the graphite onto a large surface area of the paper under the same conditions. Soft graphite is the primary material in a 5B pencil, and this was used to coat the graphite layer onto the surface of the paper. However, the GOP substrate was formed with an excessive amount of graphite debris remaining on the surface. Therefore, the debris was removed using a high-pressure nitrogen gun. This treatment makes it possible for the Pd thin film to achieve high-quality adhesion to the surface of GOP during the deposition process (Figure 1 (3)). Prior to Pd deposition, the GOP was cut into pieces with dimensions of 20 mm by 5 mm as the individual substrates for the H_2_ sensors. Pd metal wire with a purity of 99.95% was placed on a molybdenum boat in a vacuum evaporation system (VPC-260, ULVAC Technologies, Inc., Methuen, MA, USA). This system was used to achieve a high film density (superficial density), resulting in improvement of the mechanical properties of the Pd thin film. Pd films with two different thicknesses (8 nm and 60 nm) were deposited on the substrates under vacuum of $3.8\times 10^{-5}$ Torr and a current of 90 amperes. The deposition times for the films with thicknesses of 8 nm and 60 nm were different, and were controlled in accordance with the deposition rate. Subsequently, Pd microfiber network structures with different film thicknesses were formed on the GOP substrates following the deposition process (Figure 1 (4)). In order to compare their sensing performance, an 8 nm pure Pd thin film was also deposited on the same paper substrate without the graphite under the same evaporation conditions. Finally, silver electrodes were formed on the edges of the sensors using silver epoxy (Figure 1 (5)).

### 2.2. Sensor Measurements

**Figure 2:**
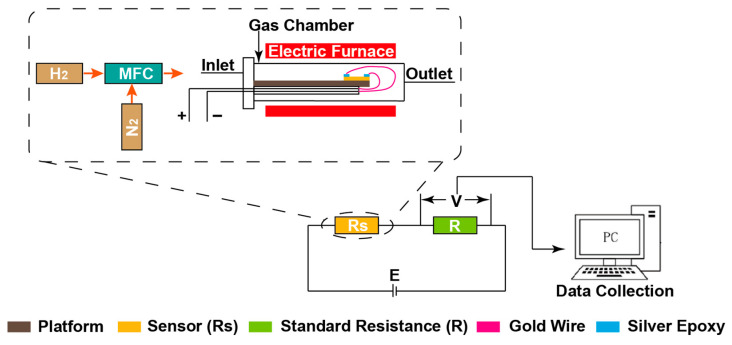
Schematic diagram of a simple H_2_ measurement circuit. The inset shows the testing system of the H_2_ sensor, including mass flow controller (MFC) and gas chamber.

The fabricated sensors with various thicknesses of Pd film were placed into a sealed quartz tube (gas chamber) for H_2_ detection and concentration measurements. Due to the resistive detection mechanism of the Pd-based H_2_ sensor, the sensor can be regarded as a variable resistor connected to a simple voltage divider circuit, as illustrated in Figure 2. A mass flow controller (MFC) was employed to control the flow rates of pure nitrogen and 1% H_2_ gas, resulting in various concentrations of H_2_ as the detection gas. H_2_ is well known to be a highly explosive gas; when its concentration reaches or is over 4% in air, a small spark can induce a violent explosion during either application or research. Therefore, for safety reasons, the maximum concentration of H_2_ was strictly controlled to be no more than 1% (10,000 ppm).

In this paper, two different thicknesses (8 nm and 60 nm) of Pd film were on GOP substrates, while one reference sample (8 nm Pd) was on a paper substrate. To obtain a reasonable evaluation of the performance for these H_2_ sensors, they had to be separately subjected to the same test steps and conditions. When the sensor was placed into the chamber, first, pure nitrogen gas was fed into the chamber for approximately 10 min in order to remove the remaining gases in the chamber. The flow rate of the mixed H_2_ gas was kept constant at 100 standard cubic centimeters per minute (SCCM) during the H_2_ testing process. To investigate the repeatability during each test, each H_2_ concentration was maintained for three cycles, and each cycle was set to a duration of 600 s. Each cycle included 300 s for the interaction of the thin film with mixed H_2_ gas, and 300 s for sensor recovery. As shown in Figure 2, based on the simple H_2_ measurement circuit, the changes in sensor resistance (Rs) with different H_2_ concentrations were converted directly into changes in the measured voltage (V), which comprised the final data that were collected. To minimize the self-heating effect during testing, the applied DC voltage source, E, was chosen to be fixed at a low voltage of 1 V. The standard resistance, R, was adjusted to be similar to the initial resistance of each H_2_ sensor, ensuring a linear sensor response. To obtain accurate data, the measured voltage was recorded using a digital multimeter once per second and logged on a computer while the sensor was exposed to each H_2_ concentration. Furthermore, the effect of the ambient temperature on the sensing performance is also considered in this paper. Hence, the H_2_ sensors were tested separately temperatures of 25 °C and 50 °C. The expression of the gas response (S) was given by

(1) $$S=\frac{R_{H}-R_{o}}{R_{o}}\times 100\%$$

where $R_{H}$ and $R_{o}$ are the sensor resistance upon exposure to H_2_ and nitrogen, respectively. All of the measurements were carried out for H_2_ concentrations of 100 ppm, 2000 ppm, 4000 ppm, 6000 ppm, 8000 ppm, and 10,000 ppm, respectively. 

## 3. Results and Discussion

### 3.1. Characterization of Pd Thin Film 

**Figure 3:**
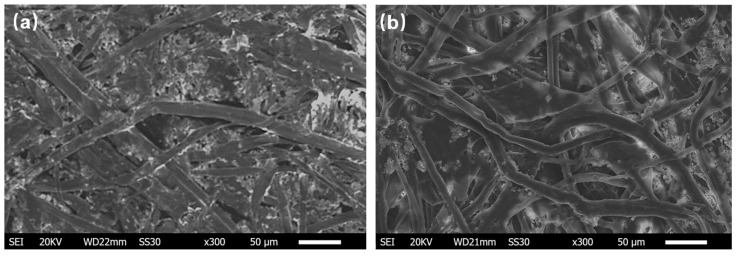
Scanning electron microscope (SEM) images of the surface of (**a**) the paper coated with graphite (GOP substrate), and (**b**) 8 nm Pd thin film deposited on the GOP substrate.

The surface morphology of the deposited Pd thin film on the GOP substrate was observed using a scanning electron microscope (SEM). The microscopic surface images of the texture detail are presented in Figure 3. 

The surface texture of paper was not damaged after graphite was coated on the paper substrate, as shown in Figure 3a. The continuous Pd microfiber network structure was formed and is clearly presented in Figure 3b, and the average diameter of the Pd microfibers was approximately 30 μm. The structural characteristics are the key factors for determining the dimensions of the materials. For instance, thin film and nanowire are defined as being two-dimensional (2D) and one-dimensional (1D) structures, respectively [14]. Based on these principles, the nanosized Pd thin film combined with the surface texture of the GOP substrate can be regarded as 1.5D (quasi-1D) structure. This low-dimensional nanostructure contributed to the improvement in H_2_ sensing performance, due to the large surface area-to-volume ratio. The detailed performance of the H_2_ sensor is further analyzed and discussed later in this section. 

### 3.2. Electrical Properties of the GOP Substrate

**Figure 4:**
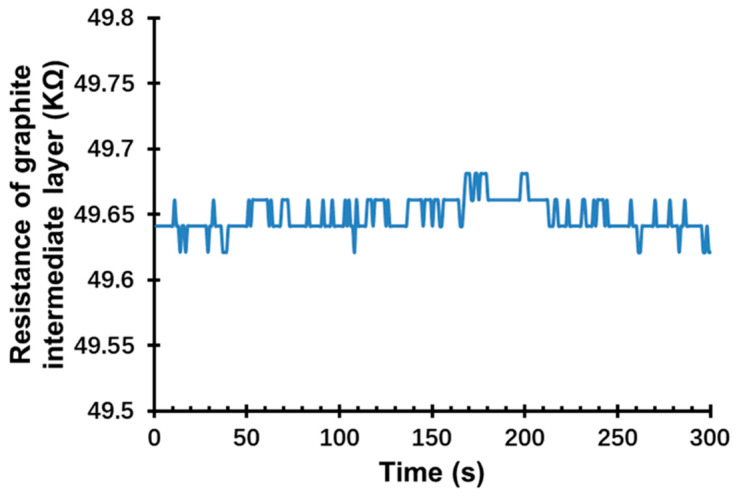
Changes in the resistance of the graphite intermediate layer when exposed to 10,000 ppm H_2_.

To verify that graphite is a suitable intermediate layer for the H_2_ sensor, the sensitivity of graphite to H_2_ was investigated. Figure 4 demonstrates that the resistance value of the GOP substrate remained steady when it was exposed to 10,000 ppm H_2_ over 300 s. Hence, the GOP substrate was confirmed to be insensitive to H_2_, which is due to the electrical properties of the graphite intermediate layer not being influenced by H_2_.

### 3.3. Discontinuity Issue in the 8 nm Pd/Paper and Graphite Intermediate Layer Solution

**Figure 5:**
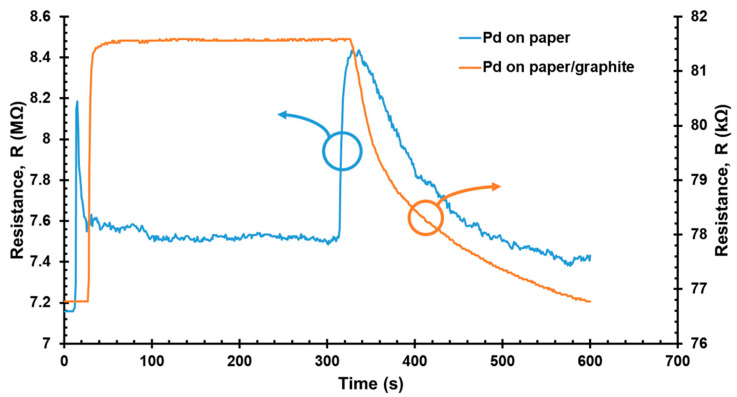
Resistance changes of 8 nm Pd on the normal photocopy paper (NPP) substrate and 8 nm Pd on the GOP substrate when exposed to 10,000 ppm H_2_ at 25 °C.

As observed in Figure 5, the resistance changes in the reference sensor (Pd on paper) resulted in an unstable detection curve, presenting two peaks when H_2_ was turned on and off. According to Figure 3, the surface morphology of the paper substrate showed high surface roughness. Therefore, after ultra-thin (8 nm) Pd film was deposited on the surface of the paper substrate, there were discontinuous microfibers coexisting with the continuous Pd microfibers. When H_2_ was turned on, initially, the resistance of the reference sensor increased sharply due to the absorption of H_2_ in the continuous Pd microfibers. However, when the Pd lattice had expanded enough to close the gaps between the discontinuous microfibers, the resistance started to decrease, as shown in Figure 5. A similar response behavior was found when H_2_ was turned off. When the absorbed H_2_ was released from Pd, the affected microfibers shrank to their initial discontinuous state, thereby reopening the gaps between the microfibers [10]. Hence, the resistance of the reference sensor increased significantly at the second peak, as shown in Figure 5. Then, the resistance decreased as a result of the release of H_2_ from the continuous Pd microfibers.

This curious response behavior has previously been reported for pure 7 nm Pd nanowire operating in N_2_ [9], it was difficult to determine the response time and gas response of the sensor on the basis of response curves like this. Although the thinner Pd film on roughness surface is able to provide a faster response and higher sensitivity, the unstable detection curve resulting from the discontinuities in the Pd microfiber is a critical issue that needs to be addressed. Therefore, an intermediate layer with high resistance and insensitivity to H_2_ was utilized to modify the interaction of Pd with the paper substrate, and to address the issue of discontinuity in the ultra-thin Pd film on the substrate. In this experimental study, graphite is proposed as an intermediate layer for the H_2_ sensor based on ultra-thin Pd film. As demonstrated in Figure 5, the sensor with 8 nm Pd on the GOP substrate exhibited smooth resistance changes, and an unsteady detection curve was not observed due to there being no discontinuous microfibers present. Hence, the low-cost and commonly available graphite can be regarded as a potential non-metallic intermediate layer for discontinuous ultra-thin Pd films on paper substrate.

### 3.4. Sensor Performance of Pd Film on GOP and NPP Substrates at Room Temperature

**Figure 6:**
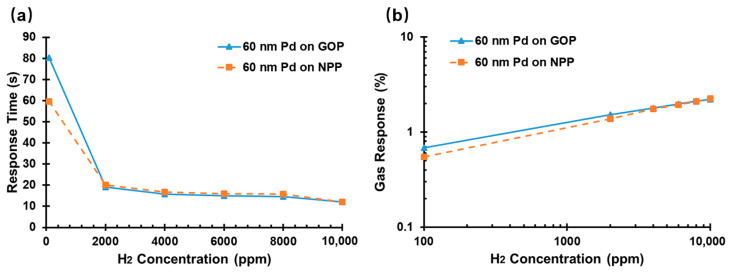
Performance of 60 nm Pd thin film H_2_ sensors based on NPP (Reprinted with permission from Ref. [15]. 2022, Springer Nature) and GOP substrate at various H_2_ concentrations at 25 °C. (**a**) Response time; (**b**) gas response.

The response time and gas response are the two primary parameters for the evaluation of H_2_ sensors. Response time is defined as the time taken to achieve 90% of the total change in sensor resistance, and the gas response can be determined using Equation (1). To investigate whether the response time and gas response of the sensor are affected by the GOP substrate, the sensing performance of the sensor comprising Pd film with a thickness of 60 nm on a GOP substrate was compared to our prior work [15], as illustrated in Figure 6.

A comparison experimental investigation was carried out to illuminate the differences in sensor resistance between the NPP and GOP substrates at 25 °C, as shown in Figure 6. Due to both sensors having the same Pd thickness, the response times showed no obvious differences between when 60 nm Pd on NPP and GOP substrates were exposed to the concentration of 2000 ppm to 10,000 ppm at 25 °C. The response times of 60 nm Pd on both substrates were equal to one another, 12 s (10,000 ppm H_2_, at 25 °C). However, the inherent texture of the paper surface was inevitably damaged by using a 5B pencil to hand paint it on paper, resulting in the surface area-to-volume ratio of the same thickness of Pd on GOP substrate being less than that on the NPP substrate. The large surface area-to-volume ratio provided fast response speed at low H_2_ concentrations. Thus, 60 nm Pd on NPP substrate appeared to have a faster response speed than on the GOP substrate when they were exposed to 100 ppm H_2_ at 25 °C, as shown in Figure 6a. As graphite is insensitive to H_2_, the degree of PdHx reaction is the same for equivalent thicknesses, and the gas responses of 60 nm Pd on NPP substrate and GOP substrate were approximately the same at 25 °C, as presented in Figure 6b. The maximum difference in gas response was smaller than 0.15% between the GOP and NPP substrates (2000 ppm H_2_, 1.51% for 60 nm Pd on GOP substrate and 1.37% on NPP substrate).

### 3.5. Temperature and Thickness Effect for Pd on GOP

**Figure 7:**
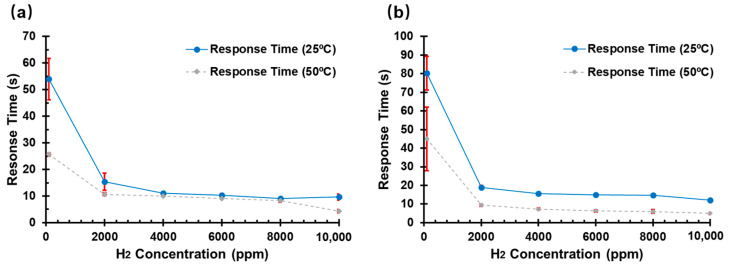
Response time (with standard deviation, sample number (N) = 3) of H_2_ sensors based on Pd thin film on GOP substrate at various H_2_ concentrations and temperatures (25 °C and 50 °C): (**a**) 8 nm and (**b**) 60 nm.

Apart from the H_2_ concentration, the sensor output is also dependent on the ambient temperature and thickness of the film, as shown in Figure 7.

At 25 °C and 10,000 ppm H_2_, the response times of the Pd-based H_2_ sensors on the GOP substrate with Pd thicknesses of 8 nm and 60 nm were 9.7 and 12 s, respectively. This phenomenon can be explained by Fick’s 1st Law, whereby a shorter H_2_ diffusion path will lead to an increase in the H_2_ diffusion flux of the Pd film at the same H_2_ partial pressure (H_2_ concentration). Therefore, the H_2_ sensor based on 8 nm Pd on a GOP substrate presented a shorter response time. The effect of temperature on the response times of the H_2_ sensors is also illustrated in Figure 7. The response time of 8 nm and 60 nm Pd to 10,000 ppm H_2_ decreased from 9.7 s to 4.3 s and from 12 s to 5 s, respectively, when the temperature was increased to 50 °C. The thermal energy increases with increasing temperature, leading to an acceleration in the movement of the gas atoms. Hence, the absorption rate and diffusivity of hydrogen atoms in Pd thin film are strongly promoted. Furthermore, the average standard deviations (N = 3) of the response time for the 8 nm and 60 nm Pd/GOP-based sensors were less than ±1% when the sensors were exposed to concentrations of 2000 ppm to 10,000 ppm, as shown in Figure 7. The Pd/GOP-based H_2_ sensors presented good repeatability when H_2_ concentration was over 2000 ppm.

### 3.6. Suppression of Heating-Induced Loss Gas Response

**Figure 8:**
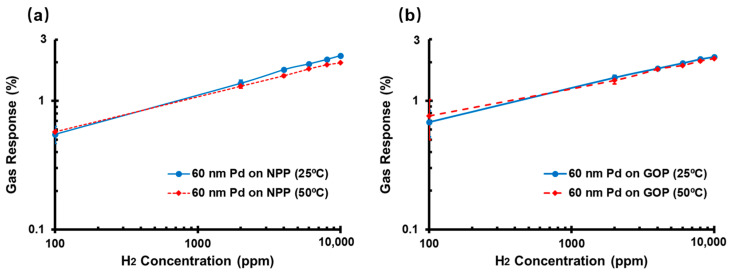
Effect of temperature on gas response for 60 nm Pd on (**a**) NPP (Reprinted with permission from Ref. [15]. 2022, Springer Nature) and (**b**) GOP substrates.

Although the response time was reduced by increasing temperature or decreasing thickness, the gas response of the Pd-based hydrogen sensor can be affected by changes in temperature [15]. As shown in Figure 8a, the gas response of 60 nm Pd on the NPP substrate decreased at elevated temperature, which was due to the reduced solubility of H in Pd with the increased temperature.

By contrast, the temperature has less impact on gas response for 60 nm Pd thin film on the GOP substrate, as presented in Figure 8b. The gas response of the H_2_ sensor based on the GOP substrate at two different operating temperatures exhibits the same variation tendency, and is not significantly affected by temperature. The gas response of the Pd thin film on the GOP substrate decreased from 2.2% to 2.1% for 10,000 ppm H_2_ at 50 °C, which is 4.5% lower than at 25 °C. Under the same conditions, the prior work indicated that the gas response decreased from 2.25% to 1.98%, which in 12% lower than at 25 °C. The gas response was affected by temperature, whether the 60 nm Pd H_2_ sensor as based on the GOP or the NPP substrate. However, due to the inverse relationship between the electrical conductivity of graphite and temperature [16], the heating-induced reduction in gas response was restrained by the reduction in the initial resistance of the sensor at 50 °C. For instance, the initial resistance of 60 nm Pd on GOP substrate decreases from 9.08 KΩ (at 20 °C) to 8.82 KΩ (at 50 °C). According to Equation (1), the initial resistance decreases with increasing temperature, and this change in resistance remains constant at 10,000 ppm H_2_, leading to increased gas response. Conversely, the influence on gas response of the solubility of H in Pd is weakened at high temperatures.

## 4. Conclusions

In this study, a simple, low-cost, and mass-producible fabrication process for 1.5D Pd-based H_2_ sensors was presented by using a paper substrate, graphite thin film, and the vacuum evaporation technique. The graphite intermediate layer on the paper substrate formed continuous 8 nm Pd microfiber networks, thereby resulting in better sensor output stability than that obtained for sensors without the intermediate layer. Furthermore, the graphite intermediate layer was confirmed to be insensitive to H_2_ and to not influence the sensing performance of the H_2_ sensor. The effects of thickness and temperature on the H_2_ sensors were investigated through the fabrication of 8 nm and 60 nm Pd-based H_2_ sensors on GOP substrates. When exposed to 10,000 ppm H_2_ at 25 °C and 50 °C, the response times of the GOP H_2_ sensor with 8 nm Pd were 9.7 s and 4.3 s, respectively. These response times are comparable to those of 1D Pd-based H_2_ sensors [3,9,12], but the 1.5D sensors proposed in this work have much lower cost, and a simpler and faster fabrication process. Additionally, the Pd thin film H_2_ sensor based on GOP substrate demonstrated that the graphite intermediate layer was capable of limiting the influence of temperature on gas response, due to the thermoelectric effect of graphite.

## Data Availability

Not applicable.
